# Proteomic analysis of ERK1/2-mediated human sickle red blood cell membrane protein phosphorylation

**DOI:** 10.1186/1559-0275-10-1

**Published:** 2013-01-03

**Authors:** Erik J Soderblom, J Will Thompson, Evan A Schwartz, Edward Chiou, Laura G Dubois, M Arthur Moseley, Rahima Zennadi

**Affiliations:** 1 Proteomics Core Facility, Duke University Medical Center, Durham, NC, USA; 2 Division of Hematology and Duke Comprehensive Sickle Cell Center, Department of Medicine, Duke University Medical Center, Durham, NC, USA; 3 Duke University Medical Center, Box 2615, Durham, NC, 27710, USA

**Keywords:** Sickle cell disease, Mitogen-activated protein kinase ERK1/2, Red blood cell membrane, Label-free quantitation, Phosphoproteomics, Glycophorin A, Hemorheology

## Abstract

**Background:**

In sickle cell disease (SCD), the mitogen-activated protein kinase (MAPK) ERK1/2 is constitutively active and can be inducible by agonist-stimulation only in sickle but not in normal human red blood cells (RBCs). ERK1/2 is involved in activation of ICAM-4-mediated sickle RBC adhesion to the endothelium. However, other effects of the ERK1/2 activation in sickle RBCs leading to the complex SCD pathophysiology, such as alteration of RBC hemorheology are unknown.

**Results:**

To further characterize global ERK1/2-induced changes in membrane protein phosphorylation within human RBCs, a label-free quantitative phosphoproteomic analysis was applied to sickle and normal RBC membrane ghosts pre-treated with U0126, a specific inhibitor of MEK1/2, the upstream kinase of ERK1/2, in the presence or absence of recombinant active ERK2. Across eight unique treatment groups, 375 phosphopeptides from 155 phosphoproteins were quantified with an average technical coefficient of variation in peak intensity of 19.8%. Sickle RBC treatment with U0126 decreased thirty-six phosphopeptides from twenty-one phosphoproteins involved in regulation of not only RBC shape, flexibility, cell morphology maintenance and adhesion, but also glucose and glutamate transport, cAMP production, degradation of misfolded proteins and receptor ubiquitination. Glycophorin A was the most affected protein in sickle RBCs by this ERK1/2 pathway, which contained 12 unique phosphorylated peptides, suggesting that in addition to its effect on sickle RBC adhesion, increased glycophorin A phosphorylation via the ERK1/2 pathway may also affect glycophorin A interactions with band 3, which could result in decreases in both anion transport by band 3 and band 3 trafficking. The abundance of twelve of the thirty-six phosphopeptides were subsequently increased in normal RBCs co-incubated with recombinant ERK2 and therefore represent specific MEK1/2 phospho-inhibitory targets mediated via ERK2.

**Conclusions:**

These findings expand upon the current model for the involvement of ERK1/2 signaling in RBCs. These findings also identify additional protein targets of this pathway other than the RBC adhesion molecule ICAM-4 and enhance the understanding of the mechanism of small molecule inhibitors of MEK/1/2/ERK1/2, which could be effective in ameliorating RBC hemorheology and adhesion, the hallmarks of SCD.

## Background

Sickle cell disease (SCD) is a hereditary blood disorder, which comprises a class of hemoglobinopathies in which a sickling variant of the β chain of hemoglobin (HbS, containing Hbβ glu^6^→ val) is expressed. Sickle red blood cells homozygous for HbS (SS RBCs) are characterized by a panoply of abnormalities, including polymerization of deoxygenated HbS [1,2], persistent oxidative membrane damage associated with HbS cyclic polymerization [3], abnormal activation of membrane cation transports, cell dehydration [4], cytoskeletal dysfunction [5], and increased adhesion [6]. These alterations in SS RBCs lead to the complex pathophysiology associated with SCD that includes vaso-occlusion, chronic hemolysis and ischemic tissue damage [7].

Studies have suggested that oxidative [8,9] and physiological stresses [10] are two of the prominent mechanisms leading to abnormalities in SS RBCs. These stresses are thought to be propagated through alterations in normal protein phosphorylation events within complex intracellular signaling pathways which may subsequently affect protein structural stability [11,12], formation of protein–protein complexes [13,14], activation of ion transport leading to cell dehydration [15-17] and RBC adhesive function [18,19]. Several proteins involved in these pathways have been previously shown to be differentially tyrosine phosphorylated in SS RBCs compared to normal (AA) RBCs, including adducin, ankyrin 1, the actin binding protein dematin, and protein band 4.1, which stabilizes the spectrin-actin interaction [14,20].

Recently, we have shown that extracellular signal-regulated kinase (ERK1/2) is hyperactive and can be inducible in SS but not in AA RBCs, and can act downstream of the cAMP/PKA/MEK1/2 pathway [18]. ERK1/2 is a member of the large mitogen-activated protein kinase (MAPK) family of serine/threonine kinases with a known downstream target consensus motif of PX[pS/T]P [21-24]. ERK1/2 responds to stimulation by a variety of different hormones, growth factors, and insulin [18,25-27] and mediates diverse functions including modulation of proliferation, differentiation, apoptosis, migration, and cell adhesion [28-31]. Aberrations in ERK1/2 signaling have been previously reported to occur in a wide range of pathologies including cancer, diabetes, viral infection, and cardiovascular disease [32,33]. In SCD, abnormal ERK1/2 phosphorylation and subsequent activation is involved in increased phosphorylation of SS RBC adhesion molecule ICAM-4, mediating RBC adhesion to the endothelium, the phenotypic hallmark of this disease [18]. It is still unknown, however, which other erythrocyte membrane proteins might be affected by the ERK1/2 signaling, and whether these proteins contribute to the pathophysiology of SCD.

To further characterize global MEK1/2/ERK1/2-induced changes in protein phosphorylation within human RBCs, we employed a previously established label-free quantitative phosphoproteomics strategy to the plasma membrane ghosts of human RBCs [18,34].

## Results and discussion

### Label-free quantitative phosphoproteomic profiling of RBC membranes

LC-MS based quantitation of global (non-targeted) phosphorylation events directly from human RBCs in disease-affected patients has been very limited in the literature [12]. The most common analytical strategies have employed coupling two-dimensional gel electrophoresis of solubilized RBC proteins with either global ^32^P labeling or anti-phosphotyrosine detection antibodies, followed by LC-MS/MS identification of phosphoproteins from differentially expressed protein spots [12,35]. In addition to the limited number of unique treatment conditions, which could be directly compared within a single study, these previous approaches do not allow residue-specific quantitation of phosphorylation events as initial detection in changes in phosphorylation status are measured at the protein level. This is particularly problematic for proteins containing multiple sites of phosphorylation, as each could be independently modulated by different kinases or phosphatases as a function of various stimuli. In addition, different phosphorylation sites could have different effect on protein function. Although strategies such as iTRAQ, commonly used for phosphoproteomic quantitation from non-cell culture based systems, address some of these limitations, the reagents add significant cost when performing the labeling at the quantities of total protein required for phosphoproteomic analysis.

**Figure 1:**
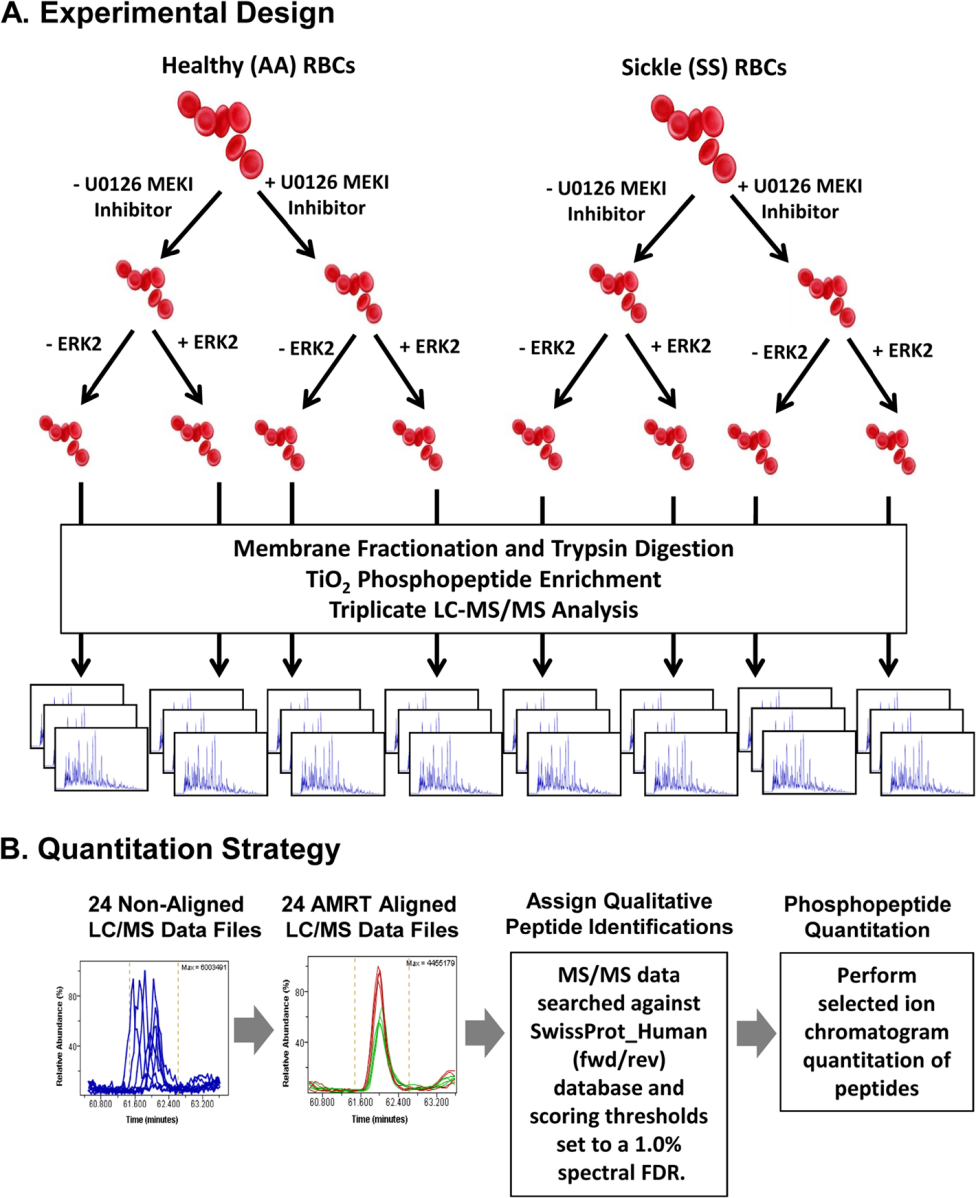
**Overview of the (A) experimental design and (B) analytical strategy of the label**-**free quantitative phosphoproteomic workflow.** RBC membrane ghosts from healthy (AA) or sickle (SS) patients were proteolytically digested and then subjected to a TiO2 based phosphopeptide enrichment. Following LC-MS/MS analysis, all data files were subjected to AMRT alignment within Rosetta Elucidator and selected ion chromatograms were generated from each phosphopeptide precursor ion to measure abundance.

To further characterize global MEK1/2/ERK1/2-induced changes in protein phosphorylation within human SS RBCs, a global label-free quantitative phosphoproteomic discovery analysis of SS and AA RBC plasma membrane ghosts was performed [18]. To determine specific involvement of the ERK1/2 activation in SS RBC membrane protein phosphorylation, each population of SS and AA RBCs was either treated or not treated with a potent MEK1/2 inhibitor (MEKI), U0126, which specifically inhibits ERK1/2 kinase activity. RBC membrane ghosts prepared from the resulting four populations of RBCs, were then either subsequently co-incubated in the presence or absence of exogenous recombinant active ERK2 (Figure 1A). Proteolytically digested membrane fractions from each of these eight unique samples were then subjected to a previously described label-free quantitative phosphoproteomics workflow utilizing reproducible TiO_2_ phosphopeptide enrichments followed by selected ion chromatographic peak quantitation of accurate-mass retention time aligned LC-MS/MS data to allow direct quantitative comparisons to be made across all treatment groups [18,34] (Figure 1B). To minimize total analysis time, each sample was analyzed in analytical triplicate by a one-dimensional LC-MS/MS analysis without any additional fractionation prior to TiO_2_ enrichment.

Across all samples, 375 unique phosphopeptides (527 total phosphorylated residues) corresponding to 155 phosphoproteins were identified at a peptide spectral false discovery rate of 1.0%. As localization of specific phosphorylated residues is critical for defining kinase specific events, all phosphopeptides were subjected to ModLoc, a probability-based localization tool implemented within Rosetta Elucidator based on the AScore algorithm [36] (Additional file 1 Figure S1A). Approximately 74% (348) of phosphorylated residues had ModLoc scores above 15 (>90% probability of correct localization), and 66% (310) had ModLoc scores above 20 (>99% probability of correct localization) (Additional file 2 Table). To assess the quantitative robustness of the label-free approach, the average technical coefficient of variation (%CV) of retention-time aligned phosphorylated peptide intensities of triplicate measurements within a treatment group were calculated (Additional file 2 Table). The mean %CV across all 375 phosphopeptides was 19.8%, with 80% of the signals having a %CVs less than 27.1% (Additional file 1 Figure S1B). The intensity of the phosphorylated peptide V173-[pY187]-R191 within the active site of ERK1/2 was used to assess inter-treatment group variation, including variation from TiO_2_ phosphopeptide enrichment, as activated ERK2 was spiked in equal amounts to four of the eight samples. The average %CV of this phosphopeptide within any treatment group was 7.0%, and across all ERK2 spiked samples was 18.1% (Additional file 3 Figures S2 A&B).

**Figure 2:**
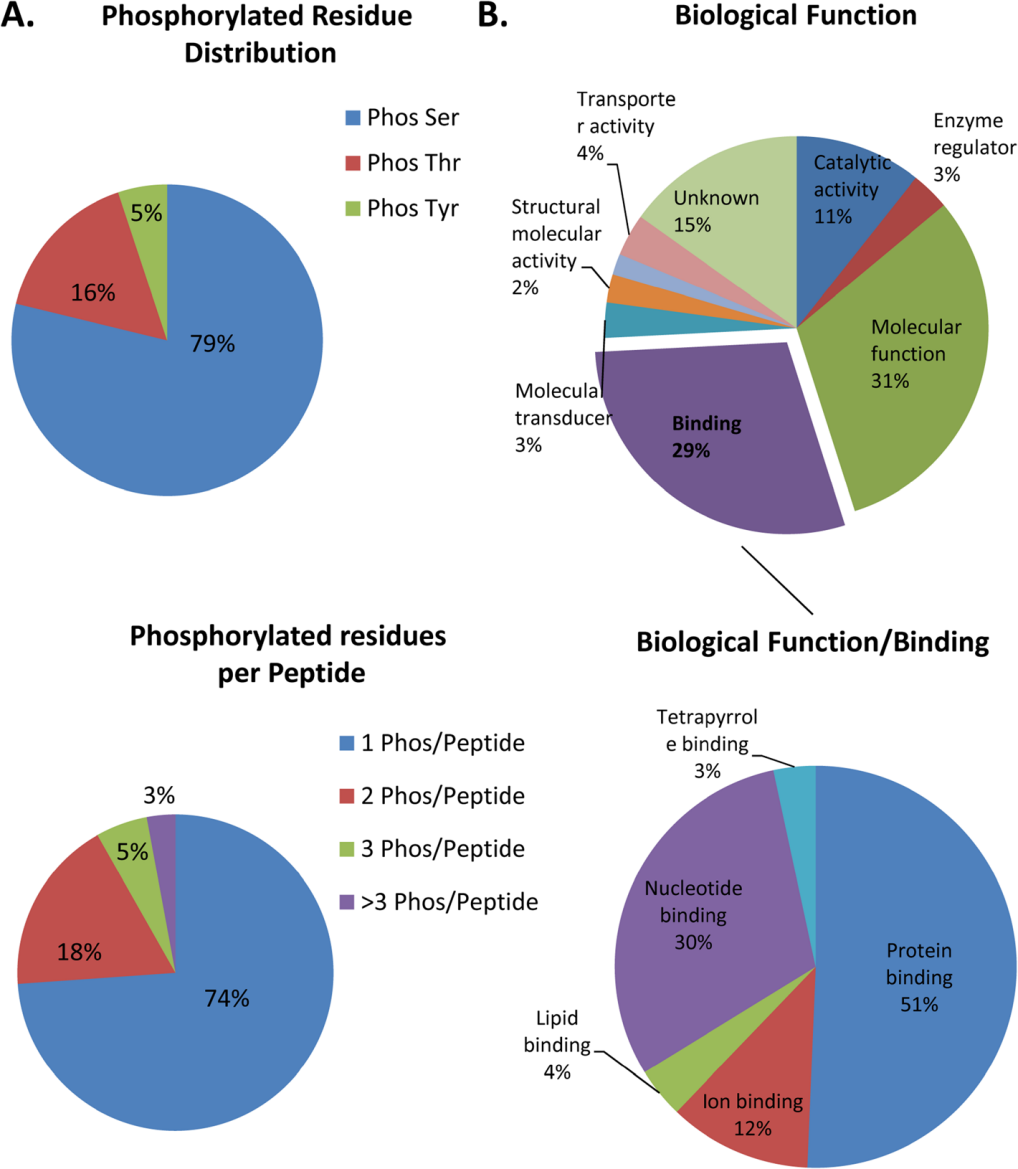
**Phosphopeptide characteristics and phosphoprotein gene ontology classification from RBC membranes.** (**A**) Distribution of pSer, pThr, and pTyr containing phosphopeptides (top panel) and number of phosphorylated residues per peptides (bottom panel) across all identified RBC membrane phosphopeptides. (**B**) NCBI Gene ontology of identified phosphoproteins implemented with Scaffold (Proteome Software).

Consistent with a majority of TiO_2_-enrichment based global mammalian phosphoproteomic studies, 79% (415) of the identified phosphorylated residues were localized to serines, 16% (85) to threonines, and 5% (27) to tyrosines, with an average of 1.4 phosphorylated residues per peptide (Figure 2A). Gene ontology classification of the biological function of the 155 identified phosphoproteins indicated nearly a third of the phosphoproteins were involved in binding as their primary biological function. Sub-classification of the binding category revealed over 80% of those phosphoproteins were involved in either protein binding (51%) or nucleotide binding (30%) (Figure 2B). Phosphoproteins involved in ion binding consisted 12% of the total phosphoproteins (Figure 2B). As these RBC samples were prepared as membrane fractions, the large number of membranous binding proteins was not unexpected. Consistent with other RBC membrane phosphorylation studies, the phosphoproteins of SS RBC membrane ghosts with the highest number of uniquely phosphorylated peptides (>10), were ankyrin-1 of the ankyrin complex, spectrin β chain of the cytoskeleton network, and proteins of the junctional complex, including α- and β-adducins, dematin and protein 4.1 (Table 1). In addition, phosphoproteins with >5 unique phosphorylated peptides, known to affect RBC shape, flexibility, anion transport and protein trafficking, and adhesion, all of which contribute to the pathophysiology of SCD, were also observed (Table 1).

**Table 1:** Highly phosphorylated proteins identified in RBC membrane ghosts

**Protein description**	**Gene name**	**Unique phosphorylated peptides**	**Unique phosphorylated residues**
Ankyrin-1	ANK1	33	23
Glyophorin A	GYPA	23	8
Alpha-adducin	ADD1	22	18
Beta-adducin	ADD2	18	11
Protein 4.1	EPB41	17	13
Dematin	EPB49	16	13
Spectrin beta chain	SPTB1	15	11
Band 3 anion transport protein	SLC4A1	14	7
Uncharacterized protein LOC388588	YA047	7	5
GTPase-activating protein and VPS9 domain-containing protein 1	GAPVD1	5	5
Lipin-2	LPIN2	5	6
Serine/threonin-protein kinase WNK1	WNK1	5	6

Twelve unique phosphoproteins were identified by at least five unique phosphopeptides. These included proteins of the ankyrin complex (ankyrin-1), the cytoskeleton network (spectrin β chain) and the junctional complex involved in binding integral membrane proteins to cytoskeletal proteins (α- and β-adducins, dematin, and protein 4.1), which affect RBC shape, flexibility and adhesion, or proteins that affect anion transport, protein trafficking and adhesion (band 3 and glycophorin A), G protein activation (GTPase activating protein), lipid biosynthesis (lipin 2) and serine/threonine phosphorylation (serine/threonine protein kinase).

### ERK1/2 Induces atypical phosphorylation of SS RBC membrane proteins

**Figure 3:**
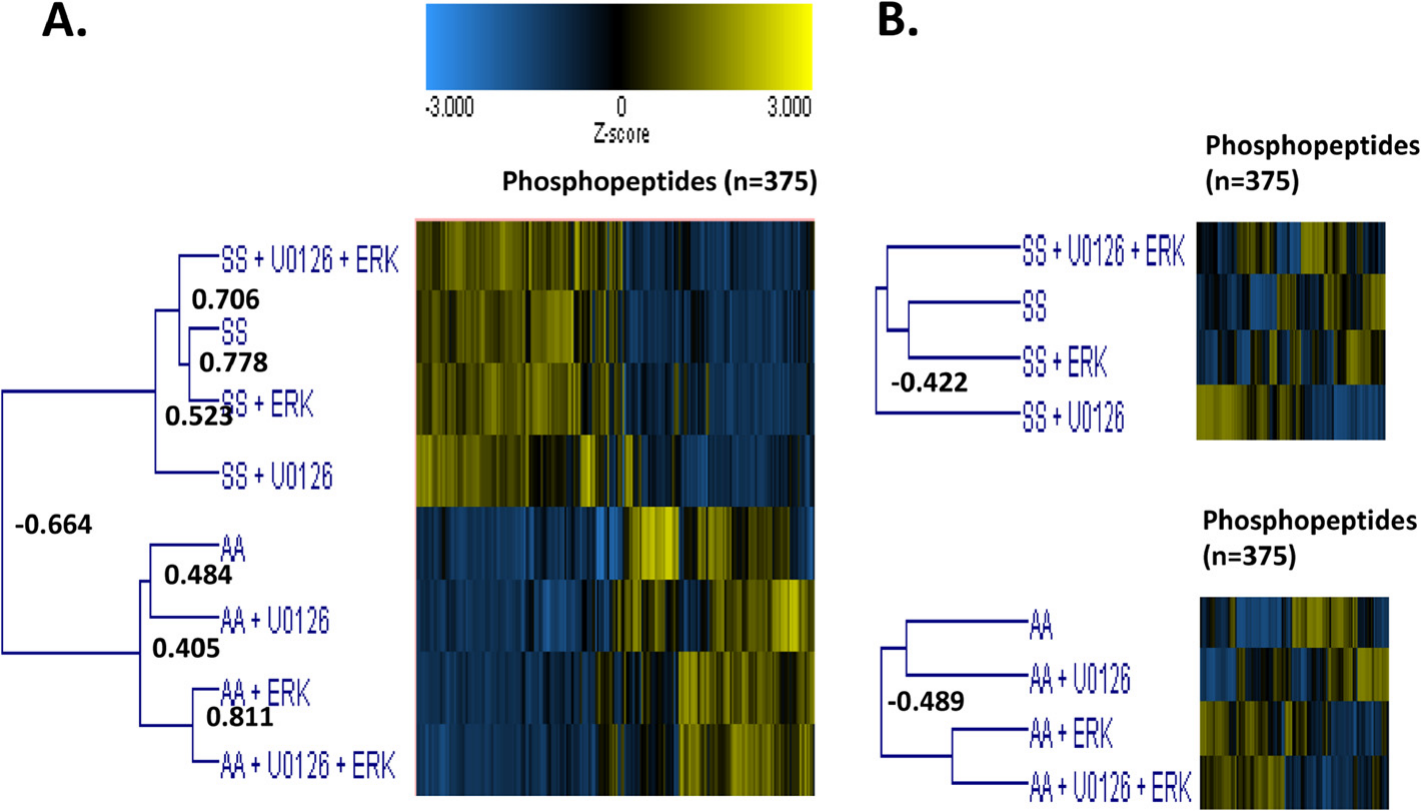
**Two**-**dimensional (2D) agglomerative cluster analysis using Z-score transformed phosphopeptide intensities across eight unique RBC treatment groups.** Person correlations were used as the measure of similarity (−1 dissimilar, +1 identical) and are shown at each branch point. (**A**) RBCs from healthy (AA) and sickle cell (SS) patients pre-treated with or without the MEK1/2 inhibitor, U0126, followed by preparation of membrane ghosts, and their subsequent co-incubation with or without activated recombinant ERK2. (**B**) Cluster analysis performed only on SS (top panel) or AA (bottom panel) RBC treatment groups.

To assess global quantitative differences between all treatment groups, data were subjected to two-dimensional agglomerative clustering using Z-score transformed (i.e. magnitude of significance of change) individual phosphopeptide intensities (Figure 3A). This analysis revealed that the most significant differentiation (most negative Pearson correlation) across all treatment groups, was the sickle versus healthy RBC phenotype, with 201 phosphopeptides being up-regulated in SS vs AA RBCs at a fold-increase of >1.75. These thresholds were chosen based on an alpha value corresponding to a 95% confidence interval in a statistical powering calculation ( http://www.dssresearch.com/KnowledgeCenter/toolkitcalculators.aspx). The weight of variation from the SS to AA RBC (−0.664) was more pronounced than the addition of exogenous active ERK2 or the inhibition of MEK1/2 activity with the MEK1/2 inhibitor U0126 (Figure 3A), suggesting that in addition to MEK1/2/ERK1/2 phosphorylation cascades in the SS RBC, other cellular signaling pathway activities may be involved. Interestingly, clustering of all phosphopeptides within only the SS RBC samples revealed the strongest differentiating factor was in the presence or absence of U0126 (Pearson correlation, -0.422) (Figures 3B, top panel), which supports the previous observation that ERK1/2 is constitutively hyperactive in these sickle RBCs and that inhibiting ERK1/2’s upstream activator, MEK1/2, alters a number of signaling events. Recovery from the U1026 treatment by addition of exogenous active ERK2 resulted in the phosphorylation profile becoming more similar to the non-treated SS RBCs (Figure 3B, top panel). In comparison, clustering of all phosphopeptides within only the AA RBC samples revealed the strongest differentiating factor was the addition of exogenous active ERK2 (−0.489) (Figure 3B, bottom panel), which is consistent with the normal inactivity of ERK1/2 in AA RBCs, and suggests that ERK1/2 signaling is indeed mediating downstream phosphorylation of a number of targets.

Putative downstream targets specific to MEK1/2-dependent activation of ERK1/2 were initially identified in SS RBCs, in which 36 unique phosphopeptides (from 21 unique phosphoproteins) decreased in abundance upon treatment with U0126. Basal ERK1/2 is already active in SS RBCs and inactive in AA RBCs [18]. Therefore, in an effort to keep the focus on the pathophysiological relevant effect of the abnormal activation of MEK1/2/ERK1/2 signaling on RBC membrane protein phosphorylation, we have presented the most physiologically relevant treatment group comparisons: AA vs SS RBCs, SS vs SS RBCs+U0126, SS vs SS RBCs+ERK2, SS+U0126 vs SS RBCs+U0126+ERK2, and AA vs AA RBCs+ERK2 in Table 2. Because label-free proteomics analysis revealed that MEK1/2/ERK1/2 signal to down-regulate 36 phosphopeptides in SS RBCs, it was important to determine the pathophysiological relevance of the abundance of these phosphopeptides by first showing that their levels were down-regulated in AA RBCs compared to SS RBCs. Comparison of individual phosphopeptide intensities between SS and AA RBCs indicates that out of these 36 phosphorylated peptides in SS RBCs, the abundance of only 25 of these phosphopeptides were decreased in AA RBCs. A negative feedback mechanism to down-regulate phosphorylation of the 25 phosphopeptides may be inactive in SS RBCs. For instance, SS RBCs have significantly higher levels of cAMP than AA RBCs [18], and PKA has been shown to exert a negative feedback loop through activation of phosphodiesterases, resulting in cAMP hydrolysis switching off downstream signaling [37]. While the MEK1/2 inhibitor U0126 was able to down-regulate these 36 unique phosphopeptides in SS RBCs, incubation of SS RBC membrane ghosts with recombinant active ERK2 in contrast, failed to increase abundance of these 36 phosphopeptides in SS RBCs (Table 2). This suggests that these peptides are already affected in SS RBCs by MEK/1/2/ERK1/2 signaling cascade, and do not necessitate further modification by exogenous ERK2. Furthermore, recombinant ERK2 was not able to fully (100%) bring up to baseline the abundance of all phosphopeptides down-regulated by U0126. As a result, 28 of these phosphopeptides did not reach the significant fold-increase of >1.75 (Table 2).

**Table 2:** **Differentially regulated phosphopeptides in SS RBCs in response to MEK1**/**2 inhibitor U0126**

**Protein description**	**Modified peptide sequence**	**AA vs SS:Fold Change(p-value)**	**SS vs SS+U0126:Fold change(p-value)**	**SS vs SS+ERK2:Fold change(p-value)**	**SS+U0126 vs SS+U0126+ERK2:Fold change(p-value)**	**AA vs AA+ERK2:fold change(p-value)**
60S acidic ribosomal protein P2	KEES*EES*DDDMGFGLFD	4.8 (7.24E-20)	−2.1 (3.61E-08)	NS	NS	NS
Adenylyl cyclase-associated protein 1	SGPKPFSAPKPQTS*PSPK	NS	−4.8 (2.00E-03)	NS	5.3 (2.00E-04)	NS
Alpha-adducin	QKGS*EENLDEAR	2.5 (2.43E-33)	−4.1 (2.80E-45)	NS	2.4 (1.25E-11)	NS
**Beta**-**adducin**	ETAPEEPGS*PAKS*APAS*PVQSPAK	**5**.**6** (**3**.**72E**-**28**)	−**1**.**9** (**3**.**87E**-**09**)^*^	**NS**	**NS**	**2**.**4** (**4**.**21E**-**06**)^*^
**Beta**-**adducin**	ETAPEEPGSPAKS*APAS*PVQSPAK	**NS**	−**1**.**8** (**1**.**80E**-**02**)^*^	**NS**	**1**.**8** (**3**.**00E**-**02**)	**1**.**8** (**2**.**00E**-**02**)^*^
Beta-adducin	TESVTSGPMSPEGSPSKS*PSK	NS	−1.9 (3.00E-02)	NS	1.9 (3.00E-03)	NS
Dematin	QPLTSPGSVS*PSR	7.9 (8.63E-05)	−4.4 (1.00E-03)	NS	NS	NS
E3 ubiquitin-protein ligase UBR4	T*SPADHGGSVGSESGGSAVDSVAGEHSVSGR	3.6 (1.55E-18)	−1.8 (1.02E-07)	NS	NS	NS
Eukaryotic translation initiation factor 4B	SQS*SDTEQQSPTSGGGK	5.6 (9.74E-06)	−2.5 (4.00E-03)	NS	2.0 (2.00E-02)	NS
facilitated glucose transporter member 1	QGGAS*QSDKTPEELFHPLGADSQV	5.0 (7.45E-10)	−3.4 (7.99E-08)	NS	NS	NS
**Glycophorin**-**A**	KS*PSDVKPLPSPDTDVPLSSVEIENPETS*DQ	**7**.**7** (**9**.**74E**-**07**)	−**1**.**9** (**2**.**10E**-**02**)	**NS**	**NS**	**2**.**0** (**5**.**00E**-**03**)
**Glycophorin**-**A**	KSPSDVKPLPS*PDT*DVPLS*SVEIENPETSDQ	**10**.**2** (**1**.**05E**-**06**)	−**2**.**3** (**5**.**00E**-**03**)	**NS**	**NS**	**2**.**1** (**8**.**04E**-**04**)
**Glycophorin**-**A**	S*PS*DVKPLPSPDTDVPLSSVEIENPETS*DQ	**6**.**3** (**9**.**08E**-**07**)	−**2**.**0** (**1**.**60E**-**02**)	**NS**	**NS**	**1**.**9** (**3**.**10E**-**02**)
Glycophorin-A	S*PS*DVKPLPSPDTDVPLSSVEIENPETSDQ	6.3 (9.08E-07)	−2.0 (8.00E-03)	NS	NS	NS
**Glycophorin**-**A**	SPSDVKPLPS*PDT*DVPLS*SVEIENPETSDQ	**7**.**4** (**8**.**28E**-**06**)	−**1**.**9** (**2**.**90E**-**02**)	**NS**	**NS**	**1**.**9** (**6**.**00E**-**03**)
Glycophorin-A	SPSDVKPLPS*PDT*DVPLSSVEIENPETSDQ	5.0 (0.00E+00)	−2.0 (2.13E-07)	NS	NS	NS
Glycophorin-A	SPSDVKPLPSPDT*DVPLS*SVEIENPETSDQ	5.0 (0.00E+00)	−1.8 (4.80E-02)	NS	NS	NS
Glycophorin-A	SPSDVKPLPSPDT*DVPLSSVEIENPETSDQ	5.0 (0.00E+00)	−2.0 (2.59E-21)	NS	NS	NS
**Glycophorin**-**A**	SPSDVKPLPSPDTDVPLS*S*VEIENPETSDQ	**5**.**1** (**3**.**49E**-**06**)	−**2**.**2** (**3**.**00E**-**03**)	**NS**	**NS**	**2**.**7** (**7**.**97E**-**05**)
Glycophorin-A	SPSDVKPLPSPDTDVPLSS*VEIENPETSDQ	NS	−1.9 (3.30E-07)	NS	NS	NS
Glycophorin-A	SPSDVKPLPSPDTDVPLSSVEIENPET*SDQ	NS	−1.9 (1.82E-13)	NS	NS	NS
Glycophorin-A	SPSDVKPLPSPDTDVPLSSVEIENPETS*DQ	NS	−1.8 (4.00E-03)	NS	NS	NS
Leucine-rich repeats and immunoglobulin-like domains protein 2	T*HPETIIALRGMNVTLTCTAVSSSDSPMST*VWR	NS	−2.9 (1.27E-23)	NS	1.8 (2.65E-05)	NS
Leucine-zipper-like transcriptional regulator 1	MAGPGST*GGQIGAAALAGGAR	88.6 (1.70E-02)	−6.5 (4.50E-02)	NS	6.7 (4.54E-06)	NS
Lipin-2	S*GGDETPSQSSDISHVLETETIFTPSSVK	3.0 (1.56E-08)	−1.9 (3.37E-04)	NS	NS	NS
**Metabotropic glutamate receptor 7**	LSHKPSDRPNGEAKT*ELCENVDPNS*PAAK	**2**.**4** (**1**.**61E**-**15**)	−**1**.**9** (**2**.**95E**-**09**)	**NS**	**NS**	**2**.**3** (**7**.**98E**-**15**)
Proteasome subunit alpha type-3	ESLKEEDES*DDDNM	2.8 (4.14E-11)	−1.8 (5.42E-06)	NS	NS	NS
Protein MICAL-2	VS*S*GIGAAAEVLVNLY*MNDHRPKAQAT*SPDLESMRK	NS	−4.1 (8.44E-05)	NS	NS	NS
Protein Wnt-16	HERWNCMITAAATTAPMGASPLFGYELS*SGTK	−2.2 (5.13E-05)	−2.0 (1.90E-02)	NS	NS	NS
**Spectrin beta chain**, **erythrocyte**	LS*SS*WESLQPEPSHPY	**3**.**5** (**4**.**17E**-**13**)	−**1**.**8** (**5**.**56E**-**05**)	**NS**	**NS**	**2**.**0** (**9**.**30E**-**11**)
Spectrin beta chain, erythrocyte	QIAERPAEETGPQEEEGETAGEAPVS*HHAATER	2.1 (6.00E-03)	−2.4 (5.77E-04)	NS	NS	NS
Transmembrane protein 151B	SPPGS*AAGES*AAGGGGGGGGPGVSEELTAAAAAAAADEGPAR	NS	−2.9 (3.91E-05)	NS	NS	NS
**U3 small nucleolar RNA**-**associated protein 15**	VVHS*FDYAAS*ILSLALAHEDETIVVGMTNGILS*VKHR	**NS**	−**2**.**9** (**7**.**39E**-**11**)	**NS**	**2**.**2** (**6**.**08E**-**05**)	**2**.**1** (**9**.**39E**-**14**)
**Uncharacterized protein LOC388588**	DGVS*LGAVS*STEEASR	**NS**	−**1**.**8** (**9**.**99E**-**05**)	**NS**	**NS**	**2**.**2** (**2**.**40E**-**09**)
Uncharacterized protein LOC388588	DGVS*LGAVSST*EEASR	2.2 (2.00E-02)	−1.8 (4.90E-02)	NS	NS	NS
**UV excision repair protein RAD23 homolog A**	EDKS*PSEESAPTTSPESVSGSVPSSGSSGR	**5**.**0** (**6**.**40E**-**38**)	−**2**.**0** (**1**.**96E**-**14**)	**NS**	**NS**	**2**.**2** (**4**.**13E**-**32**)

36 unique phosphorylated peptides were statistically down-regulated in SS RBCs upon treatment with the MEK1/2 inhibitor U0126 (SS vs SS+U0126). Of these 36, 8 phosphopeptides had their levels increased back to near basal levels upon exogenous addition of active ERK2 (SS+U0126 vs SS+U0126+ERK2). Thirteen of these phosphorylated peptides were also observed to be increased in AA RBCs upon exogenous addition of active ERK2 (AA vs AA+ERK2), suggesting specificity of downstream ERK activity. p-values < 0.05 indicate the statistically significant variances associated with differences within triplicates for that particular comparison. Biological variances associated with differences within groups are not included in these p-values. (*) indicates previously reported values [18]. NS, which refers to non-significant, indicates that a statistically significant change (based on 1.75 fold-changes and p-value < 0.05) was not measured.

We analyzed a number of these phosphoproteins referring first to the model of red blood cell membrane functional organization proposed by Anong WA et al. who identified two major protein complexes bridging the RBC membrane to cytoskeleton network: the junctional complex formed by band 3, glycophorin C, Rh group, glucose transporter, dematin, p55, adducin, band 4.1 and 4.2 with associated glycolytic enzymes and the ankyrin complex formed by band 3, glycophorin A, Rh group, ankyrin, and protein 4.2. Both complexes participate in anchoring the membrane to the actins, and α- and β-spectrins network, involving also other peripheral proteins as tropomyosin and tropomodulin [38]. Here, we found that MEK1/2-dependent ERK1/2 activation in SS RBCs affected membrane-bound proteomes of both the junctional and ankyrin complexes, including dematin, α- and β-adducins, and glycophorin A.

**Figure 4:**
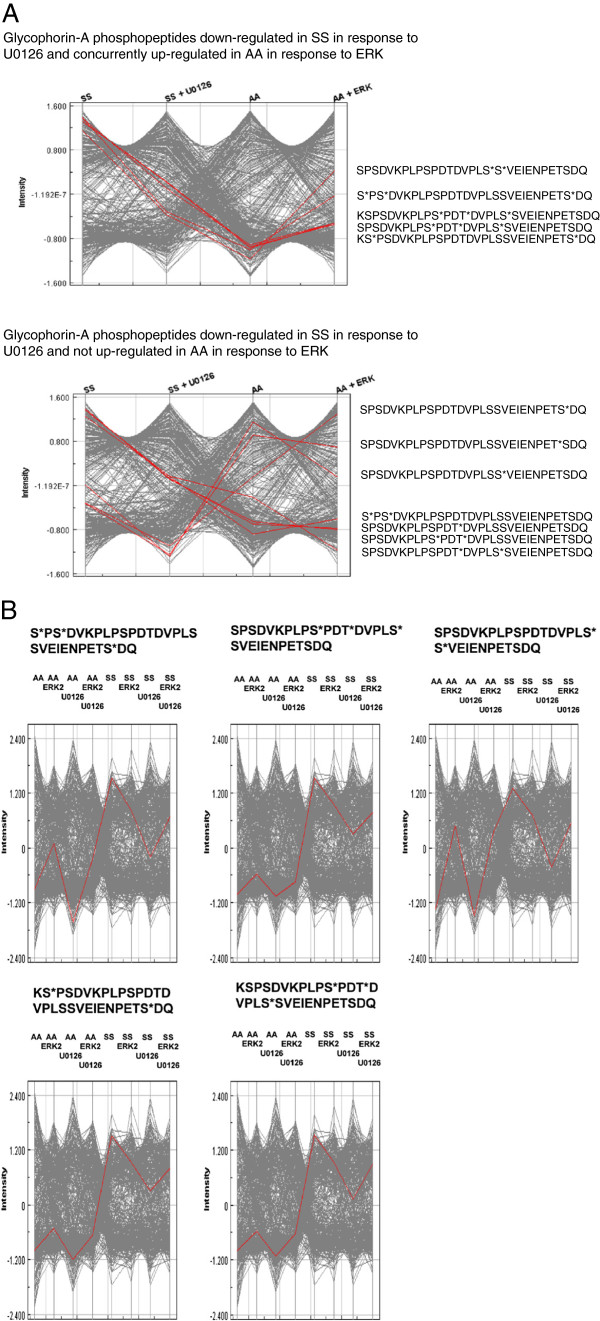
**Trend plot analysis of differentially expressed glycophorin**-**A phosphopeptides.** (**A**) Z-Score transformed phosphopeptide intensities of glycophorin-A peptides, which decreased in abundance in SS RBCs upon treatment with U0126 (indicated in Table 2) were differentiated into groups, which either did or did not increased in AA RBCs upon addition of recombinant active ERK2 to the membrane ghosts, and plotted across four relevant treatment groups. (**B**) Five glycophorin-A phosphorylated peptides, which both decreased in SS RBCs upon treatment with U0126 and increased in AA RBCs upon addition of active ERK2 to the membrane ghosts plotted across all eight treatment groups. Each data-point is plotted as an average of three technical replicates. An (*) within the peptide sequence indicates the preceding residue is phosphorylated.

Glycophorin A was the most affected protein in SS RBCs as a result of ERK1/2 activation, with 12 unique phosphorylated peptides (8 unique phosphorylated residues) being decreased in response to U0126 treatment (Table 2). The abundance of 7 of the phosphorylated residues, which were down-regulated with U0126 treatment of SS RBCs, were up-regulated in AA RBCs when exogenous active ERK2 was added to RBC membrane ghosts, suggesting that increased phosphorylation of glycophorin A by MEK1/2/ERK1/2 signaling could potentially affect SS RBC membrane properties (Table 2). To assess the changes of these phosphopeptides across the most relevant treatment groups, Z-score transformed trend plot analysis were performed and glycophorin A phosphopeptides were grouped by those which decreased in SS RBCs, and correspondingly did (top panel, 5 unique peptides) or did not (bottom panel, 7 unique peptides) increased in AA RBCs with addition of active ERK2 to the membrane ghosts (Figure 4A). For those phosphopeptides which resulted in a corresponding increase in AA RBCs with addition of recombinant ERK2, trend plot analysis were performed across all eight treatment groups (Figure 4B).

Glycophorin A, is the major sialoglycoprotein and increased SS RBC adhesion to vascular endothelial cells has been postulated to result from clustering of negatively charged glycophorin-linked sialic acid moieties at the RBC surface [6,39]. Enhanced SS RBC adhesion may also result from increased phosphorylation of glycophorin A by MEK/1/2/ERK1/2 signaling. In addition, modulation in glycophorin A phosphorylation may also affect glycophorin A interactions with band 3, which could result in decreased in both anion transport by band 3 and band 3 trafficking.

Our data also indicated that adducin-β contained three unique phosphorylated peptides, with phosphorylation of residues within the ERK1/2 consensus motif [18], suggesting that the cytoskeletal protein adducin-β is a substrate for ERK1/2 in RBCs (Table 2). A decrease in phosphorylation of these peptides was observed in U0126-treated SS RBCs, while an increase in phosphorylation was observed in both U0126-treated SS RBCs and in AA RBCs when recombinant active ERK2 was added to the membrane ghosts. However, the phosphorylated serine on either adducin-α or dematin, was not within the ERK1/2 consensus motif [18]. Phosphorylation of cytoskeletal proteins by ERK1/2 in SS RBCs may lead to cytoskeletal disorganization [14,40-43], which in turn, could potentially affect RBC adhesiveness. Our previous study has indeed shown that ERK1/2 regulates SS RBC adhesion to the endothelium [18]. Furthermore, while protein 4.1 in SS RBCs is extensively phosphorylated [14] with 17 uniquely phosphorylated peptides and 13 unique phosphorylated residues (Table 1), based on the chosen threshold fold-increase of >1.75 for this study, increased phosphorylation of protein 4.1 seems to not involve ERK1/2 signaling.

MEK1/2/ERK1/2 signaling in SS RBCs induced changes within the actins/spectrins network as well by affecting phosphorylation of β-spectrins (Table 2). Erythrocyte spectrin, the major component of the membrane skeleton, undergoes a number of naturally occurring or pathologically induced posttranslational phosphorylation via a cAMP-dependent protein kinase [44,45], which has been shown to act upstream of ERK1/2 in SS RBCs [18]. ^32^P-labeling studies indicate that only the β-subunit of spectrin is phosphorylated in intact erythrocyte [44,46-48], and an increase in β-spectrin phosphorylation by casein kinase I causes a decrease in erythrocyte membrane mechanical stability [49].

Furthermore, this analysis revealed that the peptide metabotropic glutamate receptor 7 (mGlu7) underwent serine phosphorylation at the ERK consensus motif (Table 2). In addition, a phosphopeptide detected within mGlu7 in our dataset was increased in SS RBCs by 2.4-fold compared to healthy AA RBCs. Gu et al. have also shown that mGluR7 activation occurs via an ERK-dependent mechanism, which increased cofilin activity and F-actin depolymerization [50]. mGLu7 acts as an autoreceptor mediating the feedback inhibition of glutamate release [51-53], and prolonged activation of this receptor potentiates glutamate release [54]. Changes were also observed in the status of leucine-rich repeats and immunoglobulin-like domains protein 2, leucine-zipper-like transcriptional regulator 1, and glucose transporter 1, but only in membrane ghosts prepared from SS RBCs treated with U0126 or after addition of exogenous active ERK2 to these membrane ghosts (Table 2). Changes in the status of these proteins by MEK1/2/ERK1/2 signaling may potentially disturb degradation of misfolded glycoproteins and receptor ubiquitination, and affect protein transcription. Similarly, phosphorylation of adenylyl cyclase-associated protein 1 (CAP1), which was more abundant in SS vs AA RBCs, increased via the ERK1/2 signaling only in SS RBCs [18]. CAP1 is known to regulate adenylate cyclase activation to increase cAMP levels under specific environmental conditions. Indeed, basal cAMP levels are much higher in sickle than in healthy RBCs, and cAMP/PKA can regulate ERK1/2 activation in SS RBCs [18]. CAPs are also involved in actin binding, SH3 binding, and cell morphology maintenance as well [55,56]. The failure of recombinant active ERK2 to significantly up-regulate the abundance of the phosphorylated peptides, leucine-rich repeats and immunoglobulin-like domains protein 2, leucine-zipper-like transcriptional regulator 1 and CAP1 in healthy RBCs suggests a negative regulatory mechanism might exist in these cells to prevent activation of ERK1/2-dependent phosphorylation of these membrane proteins, such as the ability of PKA to negatively affect phosphodiesterases to switch off downstream signaling [37].

### ERK1/2 Signaling highly affects phosphorylation of glycophorin A

**Figure 5:**
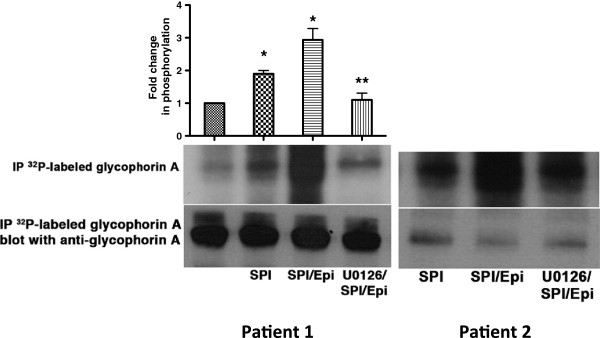
**ERK1**/**2 signaling up-regulates glycophorin A serine phosphorylation.** Inorganic ^32^P radiolabeled intact SS RBCs were incubated in the absence (patient 1: lane 1) or presence (patients 1: lanes 2, 3 and 4; and patient 2: lanes 1, 2 and 3) of serine/threonine protein phosphatase inhibitors (SPI), not followed (patient 1: lanes 1 and 2; and patient 2: lane 1) or followed by treatment with epinephrine (epi) (patient 1: lanes 3 and 4; and patient 2: lanes 2 and 3). In lane 4 (patient 1) and lane 3 (patient 2), SS RBCs were preincubated with MEK1/2 inhibitor U0126 prior to treatment with SPI followed by epi treatment. The Fold change in phosphorylation is representative of three different experiments, calculated after subtraction of cpm present in a lane (not shown) containing immunoprecipitates using immunoglobulin P3 from cpm obtained using anti-glycophorin A mAb for immunoprecipitation under each set of conditions indicated. *: *p*<0.05 and *p*<0.001 for SPI-treated and SPI+epi-treated vs. sham-treated, respectively; **: *p*<0.001 compared to SPI+epi-treated SS RBCs. Total glycophorin A loaded in each lane was detected using nitrocellulose membranes of phosphorylated glycophorin A blotted with anti-glycophorin A mAb.

The pharmacological stress hormone epinephrine can increase ERK1/2 activation in SS RBCs [18]. Because our discovery proteomics data indicated that the MEK1/2 inhibitor U0126 down-regulated the phosphorylation state of glycophorin A in a number of unique residues, we determined the contribution of activation of MEK1/2-dependent ERK1/2 signaling in glycophorin A phosphorylation in intact SS RBCs. SS RBCs were therefore treated with epinephrine to increase activation of ERK1/2 in the presence or absence of the MEK inhibitor U0126 prior to immunoprecipitation of glycophorin A. PhosphorImager analysis of immunoprecipitated ^32^P-radiolabeled glycophorin A and negative control immune complexes showed that glycophorin A of non-stimulated SS RBCs (Figure 5, lane 1, patient 1) is modestly phosphorylated at baseline, which confirms our phosphoproteomics data (Table 2). Treatment of SS RBCs with serine phosphatase inhibitors (SPI) (lane 2, patients 1; and lane 1, patient 2) slightly increased glycophorin A phosphorylation by 1.9±0.1-fold (*p*<0.05, n=3), suggesting that increased glycophorin A phosphorylation is a result of serine phosphorylation, as tyrosine phosphatase inhibitors were not present. Epinephrine in the presence of SPI had a stronger effect on glycophorin A phosphorylation (2.93±0.35-fold increase over sham-treated SS RBCs; *p*<0.001) (lane 3, patient 1; and lane 2, patient 2). However, treatment of SS RBCs with the MEK/12 inhibitor U0126 significantly decreased the combined effect of epinephrine and SPI on glycophorin A phosphorylation (lane 4, patient 1; and lane 3, patient 2) compared to cells treated with epinephrine and SPI (*p*<0.001) (lane 3 patient 1; and lane 2, patient 2). Immunoblots of ^32^P-radiolabeled glycophorin A immunoprecipitates from stimulated and non-stimulated SS RBCs indicated that a similar amount of glycophorin A was immunoprecipitated from these cells (Figure 5). Our data strongly confirm that increased glycophorin A phosphorylation is dependent on MEK1/2-dependent ERK1/2 signaling pathway in SS RBCs. It has been suggested that glycophorin contains receptors or other surface recognition sites of the erythrocyte [57]. Although the conformation of glycophorin in the lipid bilayer is not known, it has also been suggested that the glycoproteins exist as aggregates in the membrane in order to facilitate receptor function [58]. However, we do not know whether increased phosphorylation of glycophorin A affects the state of aggregation of this glycoprotein. Recently, Shapiro and Marchesi have demonstrated that the site of phosphorylation of glycophorin is located on the C-terminal portion of the protein [59]. Indeed, localization of all identified phosphorylated resides in these data were located on the C-terminal 30 residues of the protein. It remains to be determined if phosphorylation plays a role in the formation of aggregates of the protein.

## Conclusion

To date, a quantitative LC-MS based analysis of global phosphorylation events in a membrane fraction of sickle RBCs has not been reported in the literature. Here we applied a label-free quantitative phosphoproteomic strategy to characterize signaling pathways from healthy and sickle RBC membrane fractions in the presence or absence of a specific MEK1/2 inhibitor with and without subsequent ERK2 activation. The analysis resulted in the quantitation of 375 phosphopeptides from 155 unique phosphoproteins with an average technical coefficient of variation in peak intensity of 19.8%. Gene ontology analysis revealed that nearly a third of the identified phosphoproteins are involved in binding as their primary biological function. This is of considerable importance as the primary hallmark of sickle cell disease is the ability of RBCs to abnormally interact with endothelial cells, leukocytes and platelets, and these cell-cell interactions are mediated through activation of RBC surface adhesion receptors. It is important to note that the goal of this work was to perform a discovery experiment to identify candidate proteins differentially regulated by the ERK1/2 signaling pathway (Table 2). As we knew biological replications would not be available, we addressed the replication of the most biologically relevant and novel findings (phosphorylation of glycophorin A) through additional biochemical assays on replicate patients (Figure 5).

Phosphopeptide quantitation across all eight unique treatment groups indicate that the ERK1/2 pathway activation in SS RBCs could be responsible for alteration of multiple phenotypic and functional properties of the red cell, by affecting phosphorylation of thirty-six peptides from twenty-one phosphoproteins involved in adhesion, cAMP production, anion transport by band 3 and band 3 trafficking, RBC shape, flexibility, cell morphology maintenance, glucose and glutamate transport, degradation of misfolded proteins, and receptor ubiquitination, all of which play a significant role in the complex pathophysiology of the disease. Interestingly, these data revealed that glycophorin A phosphorylation was highly differentiated between healthy and sickle RBCs, and its levels of phosphorylation were modulated by the presence of the MEK1/2 inhibitor U0126 and the presence of exogenously spiked ERK2. Glycophorin A is the major sialoglycoprotein, and increased SS RBC adhesion to vascular endothelial cells has been postulated to result from clustering of negatively charged glycophorin-linked sialic acid moieties at the RBC surface [6,39]. In addition, alteration in glycophorin A phosphorylation could subsequently result in decreases in both anion transport by band 3 and band 3 trafficking. Thus, our studies further confirm ERK1/2 as a potential therapeutic target to ameliorate multiple functions of the sickle red cell, including adhesion [18] and vaso-occlusion [10,18], chronic hemolysis and ischemic tissue damage [7], all of which are associated with the pathophysiology of SCD. Finally, follow-up validations will be addressed on additional physiologically relevant molecules presented in Table 2, such as cytoskeletal proteins, and the effect of their phosphorylation by ERK1/2 on RBC function.

## Materials and methods

### Collection, preparation and treatment of RBCs

Human SCD patients homozygous for hemoglobin S were not transfused for at least three months, had not experienced vaso-occlusion for three weeks and were not on hydroxyurea. Blood samples from SCD patients and healthy donors collected into citrate tubes, were used within less than 24 h of collection. Packed RBCs were separated as previously described in detail [60]. Packed RBCs were analyzed for leukocyte and platelet contamination using an Automated Hematology Analyzer K-1000 (Sysmex, Japan). For proteomics studies, aliquots of packed RBCs were treated at 37°C for 1 h with 10 μM MEK1/2 inhibitor U0126 dissolved in dimethyl sulfoxide (DMSO). Sham-treated RBCs were incubated with the same buffer and vehicle, but without the active agent. Normal RBCs were used as controls.

### MAP kinase activity assay

Treated packed normal and SS RBCs were lysed at 4°C with lysis buffer (10 mM EDTA, 20 mM Tris, 110 mM NaCl, pH 7.5) containing 2 mM PMSF, 1% Triton X-100, phosphatase inhibitor cocktail (Sigma-Aldrich, St. Louis, MO) and protease inhibitor cocktail (Sigma). RBC membrane ghosts were then incubated with or without recombinant active human ERK2 (Sigma) at 8.2 μg/ml with a specific activity of 700 nmole/min/mg, in the presence of inhibitors of PKA, PKC, Ca^2+^/calmodulin-dependent kinase and p34^cdc2^ kinase to prevent nonspecific protein phosphorylation by these enzymes [61], and with ATP as a phosphate donor with equal membrane ghost protein amounts per assay condition. For the negative control, an equal volume of water was substituted for ATP. The reaction mixture was incubated for 20 min at 30°C. To stop the enzymatic reaction samples were placed on ice.

### RBC membrane ghost preparation and phosphopeptide enrichment

Non-radiolabeled RBC membrane ghosts isolated from packed RBCs sham-treated or treated with U0126 and incubated with or without recombinant ERK2, were spun at 14,000 rpm for 15 min at 4°C to pellet membranes. Membrane pellets were washed with 1 mL 50 mM ammonium bicarbonate (pH 8.0) with vortexing and were then spun at 14,000 rpm for 30 min at 4°C. The supernatant was then removed and 500 μL of 0.2% acid-labile surfactant ALS-1 (synthesized in house according to Yu et al. [62]) in 50 mM ammonium bicarbonate (pH 8.0) was added. Samples were subjected to probe sonication three-times for 5 sec with cooling on ice between and insoluble material was cleared by centrifugation at 14,000 rpm for 30 min at 4°C. Samples were normalized to approximately 2 μg/μl following a micro-Bradford assay (Pierce Biotechnology, Inc), and were reduced with a final concentration of 10 mM dithiothreitol at 80°C for 20 min. Samples were then alkylated with a final concentration of 20 mM iodoacetamide at room temperature for 45 min and trypsin was added to a final ratio of 1-to-50 (w/w) enzyme-to-protein and allowed to digest at 37°C for 18 hr. To remove ALS-1, samples were acidified to pH 2.0 with neat TFA, incubated at 60°C for 2 hrs and spun at 14,000 rpm for 5 min to remove hydrolyzed ALS-1. Samples were either subjected to LC-MS analysis following a 10X dilution into mobile phase A or subjected to a TiO_2_ based phosphopeptide enriched protocol [34].

To enrich for phosphorylated peptides prior to LC-MS analysis, 1,125 μg of total digested protein from RBC ghosts were brought to near dryness using vacuum centrifugation and then resuspended in 200 μL of 80% acetonitrile, 1% TFA, 50 mg/ml MassPrep Enhancer (pH 2.5) (Waters Corp., Milford, MA). Samples were loaded onto an in-house packed TiO_2_ spin column (Protea Biosciences) with a 562 μg binding capacity pre-equilibrated with 80% acetonitrile, 1% TFA (pH 2.5). For all loading, washing, and elution steps, the centrifuge was set to achieve a flow rate of no faster than 100 μL/min. Samples were washed twice with 200 μL 80% acetonitrile, 1% TFA, 50 mg/ml MassPrep Enhancer (pH 2.5) followed by two washes with 200 μL 80% acetonitrile, 1% TFA (pH 2.5). Retained peptides were eluted twice with 100 μL 20% acetonitrile, 5% aqueous ammonia (pH 10.0), acidified to pH 3 with neat formic acid and then brought to dryness using vacuum centrifugation. Prior to LC-MS analysis, each sample was resuspended in 20 μL 2% acetonitrile, 0.1% TFA, 25 mM citric acid (pH 2.5).

### LC-MS/MS analysis of RBC membrane ghosts

Chromatographic separation of phosphopeptide enriched or non-enriched samples was performed on a Waters NanoAquity UPLC equipped with a 1.7 μm BEH130 C_18_ 75 μm I.D. X 250 mm reversed-phase column. The mobile phase consisted of (A) 0.1% formic acid in water and (B) 0.1% formic acid in acetonitrile. Five μL injections of each sample were trapped for 5 min on a 5 μm Symmetry C_18_ 180 μm I.D. X 20 mm column at 20 μl/min in 99.9% A. The analytical column was then switched in-line and the mobile phase was held for 5 min at 5% B before applying a linear elution gradient of 5% B to 40% B over 90 min at 300 nL/min. The analytical column was connected to fused silica PicoTip emitter (New Objective, Cambridge, MA) with a 10 μm tip orifice and coupled to the mass spectrometer through an electrospray interface.

MS was acquired on a Thermo LTQ-Orbitrap XL mass spectrometer operating in positive-ion mode with an electrospray voltage of 2.0 kV with real-time lockmass correction on ambient polycyclodimethylsiloxane (m/z 445.120025) enabled. The instrument was set to acquire a precursor MS scan from *m*/*z* 400–2000 with r = 60,000 at *m*/*z* 400 and a target AGC setting of 1e6 ions. MS/MS spectra were acquired in the linear ion-trap for the top 5 most abundant precursor ions above a threshold of 500 counts. Maximum fill times were set to 1000 ms for full MS scans acquired in the OT and 250 ms for MS/MS acquired in the linear ion trap, with a CID energy setting of 35% and a dynamic exclusion of 60 s for previously fragmented precursor ions. Multistage activation (MSA) for neutral losses of 98.0, 49.0, and 32.33 Da was enabled to enhance fragmentation of phosphorylated peptides.

### Label-free quantitation and database searching

Label-free quantitation and integration of qualitative peptide identifications was performed using Rosetta Elucidator (v 3.3, Rosetta Inpharmatics, Seattle, WA). All raw LC-MS/MS data were imported and subjected to chromatographic retention time alignment using the PeakTeller® algorithm with a minimum peak time width set to 6 s, alignment search distance set to 4 min and the refine alignment option enabled. Quantitation of all detected signals in the precursor MS spectra was performed within Elucidator following the generation of extracted ion chromatograms for each detected precursor ion. Fold-change values between treatment groups were calculated on the phosphopeptide level from the averages of the sum of all features associated with the precursor ion within a technical replicate. To account for slight differences in total peptide loading between injections, all of the features within an LC-MS analysis were subjected to a robust mean normalization of all of the feature intensities, which excluded the highest and lowest 10% of the signals.

Qualitative peptide identifications were made by generating DTA files for all precursor ions, which had associated MS/MS spectra. DTA files were submitted to Mascot (version 2.2.04, Matrix Science, Boston, MA) and searched against a *Homo sapiens* protein database downloaded from SwissProt concatenated with the sequence-reversed version of each entry (download March 2009, 20336 forward entries). Search tolerances of 10 ppm precursor and 0.8 Da product ions were applied and all data were searched using trypsin specificity with up to two missed cleavages. Static modification of Carbamidomethylation (+57.0214 Da on C) and dynamic modifications of oxidation (+15.9949 Da on M) and phosphorylation (+79.9663 Da on STY) were employed. False-discovery rate were determined by adjusting the Mascot peptide ion score threshold to allow a 1% occurrence of peptide spectral matches from reverse protein entries for phosphopeptide enriched experiments.

A tabular form of the raw data, including Protein Accession number, Protein Description, Modified Peptide Sequence, ModLoc Max Score, Mascot Ion Score, and Intensities/Standard Deviation for each phosphorylated peptide within each treatment group has been uploaded as an Additional file 2.

### Glycophorin A phosphorylation and immunoprecipitation

Packed RBCs ^32^P-labeled as previously described [63], were sham-treated, or incubated with serine/threonine phosphatase inhibitor (SPI) cocktail (Sigma) for 30 min, SPI cocktail followed by 1 min treatment with 20 nM epinephrine, or pre-incubated with 10 μM U0126 for 1 h followed by SPI cocktail, then treated with 20 nM epinephrine for 1 min. Cells were then washed 4 times. Glycophorin A immunoprecipitation using anti-glycophorin A monoclonal antibody (mAb) (Abcam, Cambridge, MA) and the negative control immunoglobulin P3, and total and phospho-glycophorin A detection were performed as previously described in detail [60]. To confirm that the immunoprecipitates were specific for glycophorin A, anti-glycophorin A mAb and the negative control P3 were used to immunoprecipitate glycophorin A from non-radiolabeled treated SS RBCs. Blots were immunostained with anti-glycophorin A mAb.

### Data clustering and statistical analysis

Global characterization of phosphoproteomic profiles across all treatment groups was accomplished using two-dimensional clustering within Rosetta Elucidator. Individual phosphopeptide intensities within a treatment group were averaged and then converted to a Z-score to measure significance of change rather than absolute change. Z-score corrected phosphopeptide intensities were then subjected to an agglomerative clustering algorithm, using an average link heuristic criteria. Pearson correlation metrics were used to define similarity, with a score of 1 being identical and −1 being completely dissimilar. *P*-values for phosphoproteomic data was calculated using a ratio error model [64]. P^32^ glycophorin-A data were compared using parametric analyses (GraphPad Prism 5 Software, San Diego, CA), including repeated and non-repeated measures of analysis of variance (ANOVA). One-way and two-way ANOVA analyses were followed by Bonferroni corrections for multiple comparisons (multiplying the *p* value by the number of comparisons). A *p*-value < 0.05 was considered significant.

## Competing interests

The authors declare no competing financial interests.

## Authors’ contributions

E.S. performed mass spectrometry separation and label-free phosphopeptide enrichment experiments, participated in the interpretation of the corresponding data, generated the graphs for proteomic data and wrote the manuscript; W.T. helped design proteomic experiments and interpret the corresponding data, and helped edit the manuscript; E.S. helped perform some of the Western blot experiments related to glycophorin A phosphorylation; E.C. helped perform the Western blot experiments related to glycophorin A phosphorylation; L.D. helped perform mass spectrometry sample preparation A.M. helped edit the manuscript; and R.Z. conceived the hypothesis, designed and led the project, prepared the samples for mass spectrometry separation and label-free phosphopeptide enrichment experiments, performed and generated the data for immunoprecipitation experiments, analyzed and interpreted the data and wrote the manuscript. All authors read and approved the final manuscript.

## Supplementary Material

Additional file 1 **Figure S1.** Phosphorylated residue localization and technical variation of phosphorylated peak intensity. (A) ModLoc site localization scoring distributions across all 375 unique phosphoryalted peptides from RBC ghost preparations. (B) Coefficient of variation (%CV) distribution of measured phosphopeptide peak intensities from triplicate LC-MS analysis of a treatment group following accurate-mass and retention time alignment. Error bars indicate standard deviation within each %CV bin across all eight treatment groups. Click here for file

Additional file 2 **Phosphopeptides identified in TiO2-enriched RBC membrane fractions.** From left to right; protein accession number, protein description, modified peptide sequence, ModLoc Max Score, Mascot ion score, and Intensities/Standard Deviation for each phosphorylated peptide within each treatment group. Click here for file

Additional file 3 **Figure S2.** Inter-treatment group variation of ERK phosphorylated peptide. Selected ion chromatogram (A) and peak quantitation (B) of 173-VADPDHDHTGFLTE[pY]VATR-191 ([M+3H]^3+^ 741.999 m/z), the active form of Mitogen-Activated Protein Kinase-1 (SwissProt_MAPK1, ERK1/2), across 24 LC-MS injections. This peptide was qualitatively identified with a maximum mascot ion score of 63.3 and a site localization ModLoc score of 41 +/− 12. Click here for file
