# Supplementary figures and images for: Proteomic analysis of ERK1/2-mediated human sickle red blood cell membrane protein phosphorylation
Source: Clin Proteomics. 2013 Jan 3;10(1):1. doi: 10.1186/1559-0275-10-1 (PMC3558407; doi:10.1186/1559-0275-10-1)

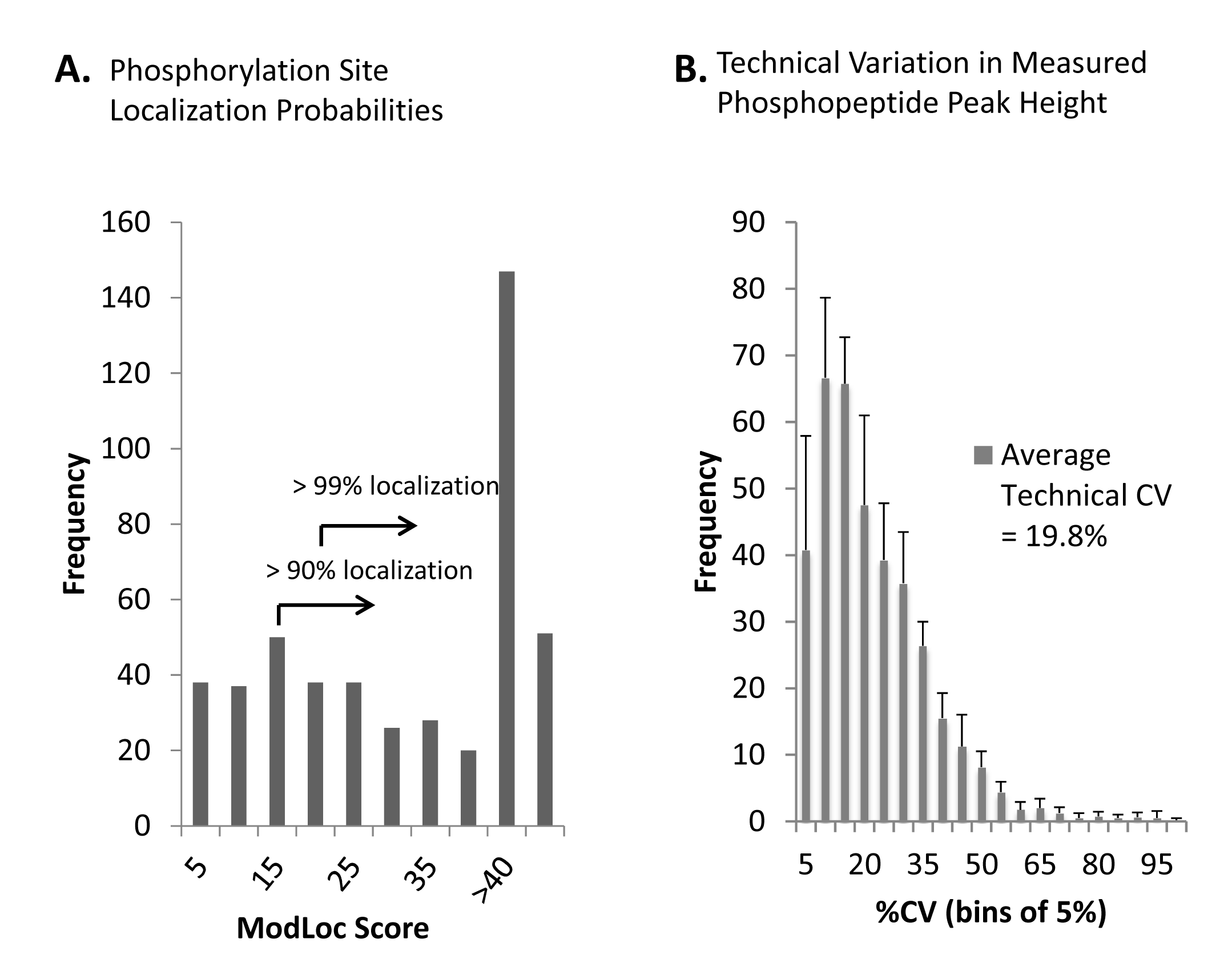

Supplement: Additional file 1 — Figure S1. Phosphorylated residue localization and technical variation of phosphorylated peak intensity. (A) ModLoc site localization scoring distributions across all 375 unique phosphoryalted peptides from RBC ghost preparations. (B) Coefficient of variation (%CV) distribution of measured phosphopeptide peak intensities from triplicate LC-MS analysis of a treatment group following accurate-mass and retention time alignment. Error bars indicate standard deviation within each %CV bin across all eight treatment groups. [file 1559-0275-10-1-S1.tiff]

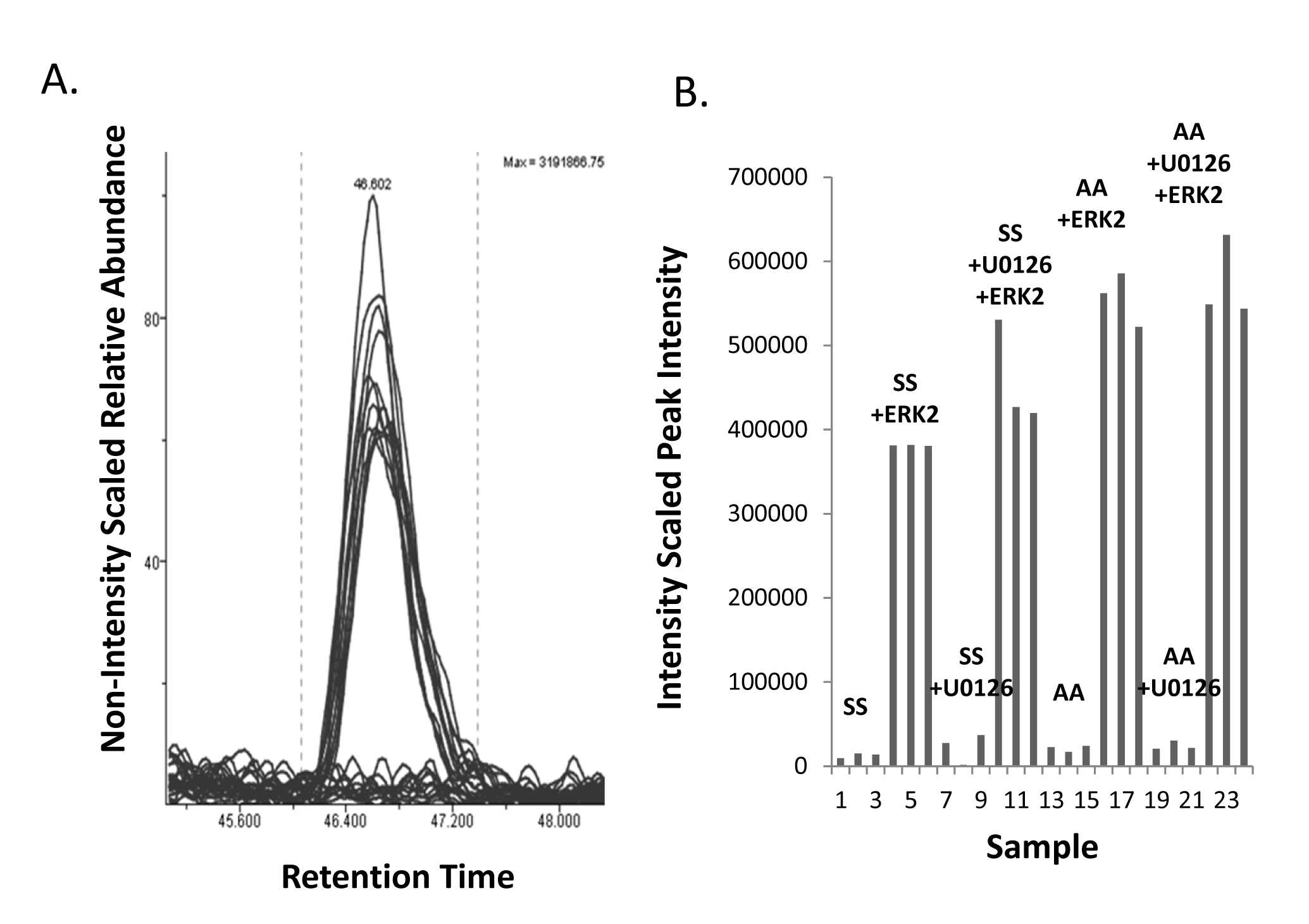

Supplement: Additional file 2 — Phosphopeptides identified in TiO2-enriched RBC membrane fractions. From left to right; protein accession number, protein description, modified peptide sequence, ModLoc Max Score, Mascot ion score, and Intensities/Standard Deviation for each phosphorylated peptide within each treatment group. [file 1559-0275-10-1-S2.tiff]
